# Integrating Genomic Data Sets for Knowledge Discovery: An Informed Approach to Management of Captive Endangered Species

**DOI:** 10.1155/2016/2374610

**Published:** 2016-06-08

**Authors:** Kristopher J. L. Irizarry, Doug Bryant, Jordan Kalish, Curtis Eng, Peggy L. Schmidt, Gini Barrett, Margaret C. Barr

**Affiliations:** ^1^College of Veterinary Medicine, Western University of Health Sciences, 309 East Second Street, Pomona, CA 91766, USA; ^2^The Applied Genomics Center, Graduate College of Biomedical Sciences, Western University of Health Sciences, 309 East Second Street, Pomona, CA 91766, USA; ^3^Danforth Center, 975 N. Warson Road, St. Louis, MO 63132, USA

## Abstract

Many endangered captive populations exhibit reduced genetic diversity resulting in health issues that impact reproductive fitness and quality of life. Numerous cost effective genomic sequencing and genotyping technologies provide unparalleled opportunity for incorporating genomics knowledge in management of endangered species. Genomic data, such as sequence data, transcriptome data, and genotyping data, provide critical information about a captive population that, when leveraged correctly, can be utilized to maximize population genetic variation while simultaneously reducing unintended introduction or propagation of undesirable phenotypes. Current approaches aimed at managing endangered captive populations utilize species survival plans (SSPs) that rely upon mean kinship estimates to maximize genetic diversity while simultaneously avoiding artificial selection in the breeding program. However, as genomic resources increase for each endangered species, the potential knowledge available for management also increases. Unlike model organisms in which considerable scientific resources are used to experimentally validate genotype-phenotype relationships, endangered species typically lack the necessary sample sizes and economic resources required for such studies. Even so, in the absence of experimentally verified genetic discoveries, genomics data still provides value. In fact, bioinformatics and comparative genomics approaches offer mechanisms for translating these raw genomics data sets into integrated knowledge that enable an informed approach to endangered species management.

## 1. Introduction

Today's technology makes it feasible to sequence the genome of almost any species of interest and to investigate complex genetic relationships in populations of animals [1–3]. However, the task of translating enormous amounts of genetic data into practical applications is still a work in progress [4–6]. As the ability to predict the functional impact of genetic variations within a population improves, genomics data could provide a powerful tool for informing the management of captive endangered species.

Whole genome sequencing has the potential to be very effective in conservation efforts [2]. In the past, genetic studies have focused on a few loci from many individuals; genomics now allows the focus to switch to many genes from a few individuals. This is particularly important when studying endangered species and working with very limited sample sizes. In addition, genomics provides us with the opportunity to look at the entire genome and not just the pieces that directly code for proteins in attempts to better understand if they contribute to survival. Overall, genomics has the opportunity to play a pivotal role in proactively understanding pressures and potential stressors that are leading some species into extinction [7].

Endangered species can benefit greatly from the use of comparative genomics. Using the genome sequence from close relatives as reference, the genome of endangered species can be compiled with relatively few samples, which is particularly important when only a limited number of animals exist in captivity. Once the genome is sequenced, identifying deleterious mutations caused by single nucleotide variants (SNVs) provides a valuable resource for management of captive populations [8]. For example, the California condor diverged from the chicken over 100 million years ago but comparison of the genomes reveals useful information in the management of this endangered species. A BAC library was created for the condor and comparative genomics was employed, identifying 93 genes that were aligned with the chicken chromosome map [9]. Using this approach, several genes involved in bone and cartilage formation have been identified, and further tests may reveal the role these genes play in the condor chondrodystrophy, a heritable embryonic lethal phenotype that is present in the population. Understanding the genetic basis of this disease greatly increases the ability to manage and progress the care of this captive population [10]. Another finding in these birds is a mutation in one of the estrogen receptors that has been linked to altered receptor activation by endocrine-disrupting chemicals, such as dichlorodiphenyltrichloroethane (DDT) and its metabolites. This mutation may affect their sensitivity to such chemicals, resulting in reproductive disorders [11]. A comparative genomics approach could be used to identify additional genes associated with reproduction that might also be impacted by environmental contaminants and other factors, providing a multifaceted view of reproductive issues in these animals.

Major histocompatibility complex (MHC) genes are the most polymorphic genes in the vertebrate genome and can be helpful markers in identifying population diversity as well as indirectly measuring immunologic fitness. Ujvari and Belov [12] emphasize the use of markers like MHC to better plan conservation and manage captive populations. For example, MHC has been implicated in mate choice and pregnancy outcomes, which could be very important when working with captive breeding programs. The MHC class II B polymorphism was investigated in a number of wild populations of gorilla via noninvasive fecal sample collection coupled with next-generation sequencing [13]. The analysis led to the identification of 18 different alleles that had not been previously characterized in the gorilla populations. MHC genes have been identified as markers for immune function and survival in almost all vertebrates, and genetic variation of this region can lead to increased or decreased disease susceptibilities [14, 15].

Genome sequencing has enabled huge progress in understanding disease even in very understudied subjects. The Tasmanian devil has recently suffered a major population crash from the result of devil facial tumor disease, and genomics approaches have been implemented to further understand the specifics of the disease and potentials for more effective treatments [16]. Hawaiian honeycreepers are an adaptive radiation that contains numerous endangered species and is of particular interest due to their diverse phenotypes and heterogeneous responses to avian malaria. Genome sequencing and assembly with SNP discovery are providing insight into why some of these birds are malaria-resistant and others are not [17].

In attempts to preserve some of the most endangered species, zoos and other organizations have established captive breeding programs and in some cases reintroduced endangered species back into the wild. Genomics has the potential to facilitate assessing the genetic fitness of individuals within a population and assist programs in correctly identifying the most successful breeding pairs to ensure genetic diversity among future generations. Identifying the best founder individuals in a captive breeding program can greatly increase the success of the program and potentially enhance fitness of the species [18–21].

## 2. Types of Genomics Data and Analyses

A variety of genomics data types exist. Genomic sequence data provides the foundation of an annotation framework anchored to primary nucleotide sequence organized around contigs and scaffolds comprising individual chromosomes. Sets of sequence intervals corresponding to genes, exons, introns, promoters, enhancers, untranslated regions (UTRs), and intergenic regions comprise the organizational structure of genomics sequence data. Protein coding gene level annotation is structured around the one-to-many relationship that potentially exists between genomic loci and their paired RNA transcripts and protein sequences. Together, the set of transcripts derived from a single genomic locus represents the sequence complexity of gene expression. Additional levels of complexity arise through the tissue and cell specific patterns of expression associated with each transcript as well as the combination of cis regulatory elements responsible for both the transcript sequence and expression patterns. Transcriptome sequencing can provide a wealth of information about the pattern of gene expression including the alternative isoforms, protein coding single nucleotide polymorphisms (SNPs), and tissue specific patterns of expression important in development, health, and disease [22].

Genetic variation is an integral component of genomics data and represents the ability to investigate relationships that may exist between genes, tissues, individuals, and phenotypes [23]. Among the genetic variants that exist are repetitive elements, copy number variations, single nucleotide substitutions, single nucleotide insertions/deletions (indels), genomic inversions, and genomic duplications, to name a few. The most prevalent variants are SNPs, and once a sizable set has been identified, relationships between allelic variation and phenotypes can be empirically investigated.

Traditionally, genotyping in conservation genetics was accomplished using individual assays targeting a single polymorphism, such as restriction fragment length polymorphism (RFLP) analysis [24]. As the number of polymorphisms assayed increased, PCR-based methods of genotyping replaced the more cumbersome RFLP methods. In parallel, repeat polymorphisms can also be interrogated via PCR-based sequencing methods to take advantage of the increased value associated with multiallelic markers. As the number of polymorphisms under investigation approaches thousands to hundreds of thousands, nucleotide hybridization chips become the platform of choice [25]. Other methods, such as genotyping by sequencing (GBS) [26] and SNP analysis via transcriptome sequencing [27], offer opportunities not only to genotype known polymorphisms, but also to detect de novo and private variants that are unique to a specific individual. Sequencing methods of polymorphism discovery have the potential to identify hundreds of thousands to millions of SNPs, depending upon the depth of sequencing coverage and the relative extent of genetic diversity represented in the samples [28].

Over the past decade, genomic data acquisition has become increasingly routine. Since the year 2005, data output from next-generation sequencing (NGS) platforms has more than doubled each year [25]. Whole human genomes worth of sequence data can be produced as cheaply as $1000, a staggering decrease from the original human genome project's cost of nearly $3 billion, and produced over days or hours instead of what was originally many years [29]. NGS technology has enabled a multitude of scientific investigations that previously could never have even been considered.

Genomics analyses in general, including comparative genomics studies, depend heavily on having an available high-quality genome reference sequence. Unfortunately, unlike model organisms such as mouse, endangered species typically lack significant or any available genomic resources [3]. An important first step in genomics-enabled endangered species management is, therefore, development of a high-quality genome reference sequence. While it is not an insignificant undertaking, reference genome sequence development is becoming more and more commonplace.

**Figure 1:**
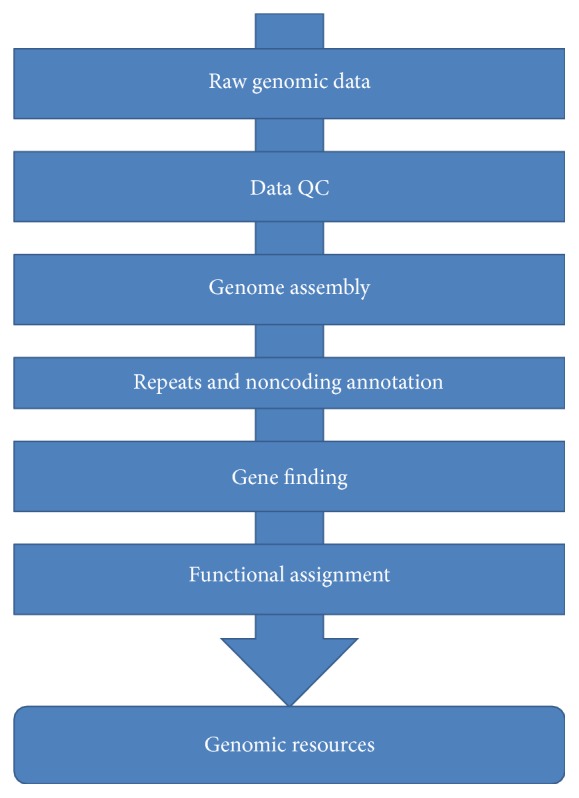
Overview of genomic resource development steps. Raw data is quality controlled and then assembled. Repeats and noncoding features are structurally annotated and used to mask the genome sequence. Genes are then called on this masked sequence, followed by functional assignment. Genome assembly, repeats, noncoding features, and genes constitute basic genomic resources.

Reference genome sequence development starts with raw sequence data (Figure 1). While reference sequences of large complex eukaryotic species constructed entirely from single molecule sequencing technology is becoming more common [30], typically such projects are largely based on paired-end and mate-pair data from NGS platforms. Paired-end data results from sequencing the paired-ends of inserts in the range of hundreds of base pairs, while mate-pair data results from sequencing the ends of inserts in the range of thousands of base pairs. Sufficient coverage from both library types is imperative for the resulting genomic reference sequence to be of sufficient quality. These genome sequence data are then processed and assembled [31] using appropriate methods based on actual data types, with the resulting assembly constituting the species draft genome sequence.

Once a draft genome sequence is constructed, structural annotation can proceed (Figure 1). Here, features such as genomic repeats, genes, and noncoding RNAs are identified, a process that can be informed by closely related model organisms. For example, the well-annotated mouse and human genomes and structural annotations can be used to help identify genes in mammalian endangered species, resulting in higher quality gene and other feature calls than what would be available without such additional information. Genomic data from more closely related species (e.g., domestic bovine genome for endangered bovine species) can be used, if available [32]. Typically, repeats are first identified and used to mask the draft genome sequence, a step necessary to help reduce false positive gene calls resulting from transposable elements and other nongene features. Noncoding RNA molecules are also identified, generally with homology- and motif-based methods. Genes are then identified on the repeat masked genome sequence, informed by closely related model organismal sequence, and, if transcriptome data from the target endangered species is available, these transcript data as well. Once genes have been identified, functional assignments can be made via homology to related species functional annotation, homology versus compiled databases such as the NCBI NR database (http://www.ncbi.nlm.nih.gov/refseq/), the Uniprot database (http://www.uniprot.org/), Conserved Elements from Genomic Alignments (CEGA, http://cega.ezlab.org/) [33], and motif-based methods such as those leveraged with InterProScan (http://www.ebi.ac.uk/Tools/pfa/iprscan5/).

These genomic resources, including the (1) draft genome sequence, (2) structural annotation, and (3) functional annotation, form the basis of all subsequent genomic analyses (Figure 1). Whereas a high depth of sequencing is required in development of the draft genome sequence itself, comparatively very low coverage is all that is needed to assess genomic traits of further individuals. While Illumina recommends 30x coverage for accurate single nucleotide polymorphism (SNP) and short insertion and deletion (indel) identification, it is typical to perform such analyses using much lower coverage on the order of 10–15x. It is common to sequence tens of individuals together (provided with identification labels such as multiplex identifiers, MDs) in a single sequencing unit and then bioinformatically separate by individual and analyze the resulting sequence data on a per-individual basis [34]. Such methods form a powerful mechanism on which we analyze large numbers of individuals for very low cost. A per-individual and practically exhaustive genomic fingerprint, or the identification of all SNPs and indels in each individual, allows direct comparison between all available members in an endangered species population [35–37].

## 3. Comparative Genomics Approaches

Just as comparative physiology and comparative anatomy offer a context for appreciating the mechanisms underlying variation in form and function, comparative genomics aids in elucidating conserved and divergent genetic mechanisms associated with specific phenotypes. Comparative genomics methods represent undervalued, yet extremely powerful tools for exploring patterns of shared and divergent biology between pairs of organisms' genomes [38]. Unlike well-studied model organisms, such as mouse and dog, captive endangered species are relatively poorly studied, and the possibility of developing knockouts or transgenic strains of endangered species expressly for experimental exploration of their biology is not a feasible option. Subsequently, the rate at which functionally important genomic signals are identified in endangered species lags significantly behind the rate for typical model organisms. In fact, sometimes genomics sequence data provides relatively little benefit for endangered species conservation efforts, even well after the genomics resources have been produced and deposited into a public database/repository. This is not a consequence of poor genomic quality, but rather the challenge in translating the raw genomic data into management informing knowledge.

Of particular interest are comparative genomics approaches that can rapidly identify functionally important genomic regions with implications for health and disease. The mouse is one of the most widely studied genetic models in the world, resulting in the production of mouse lines having mutations in over one-third of the genes encoded in the mouse genome [39]. The International Mouse Phenotyping Consortium (IMPC) is a collaborative functional genomics effort between laboratories in America, Germany, United Kingdom, France, Canada, China, and Japan. The IMPC has characterized phenotype data for approximately 2000 mouse genes and plans to have a total of 5000 genes characterized by 2016 [40]. The Mouse Genome Database (MGD) is a central repository for mouse functional genomics data and resources including phenotype annotations for mouse genes. Functional annotation in the form of ontologies, such as the Gene Ontology and Mammalian Phenotype Ontology [41], is integrated with the mouse genome.

**Figure 2:**
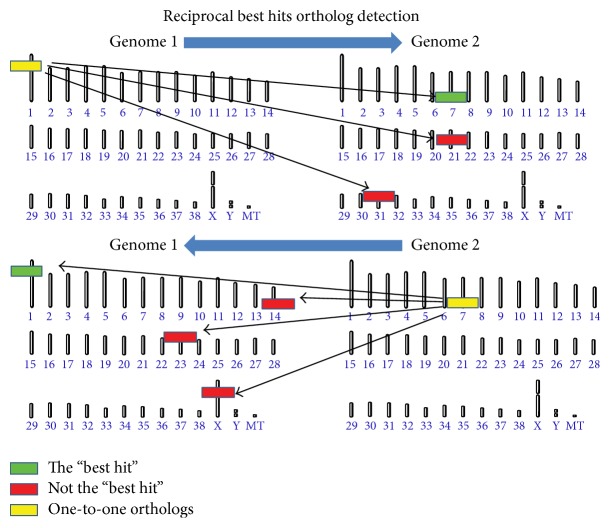
Identifying one-to-one orthologs between two genomes. The foundation of comparative genomics rests upon the ability to leverage annotation in a well-annotated genome in order to make inferences about genes in an undercharacterized genome. High sequence identity exists between orthologs (yellow genes, best hits) and paralogs (red genes, nonbest hits); however, paralogs are known to diverge in function much faster than orthologs. In order to achieve high confidence genotype-phenotype relationships via comparative genomics, it is essential to differentiate between orthologs and paralogs. Although complex relationships exist between orthologs, such as many-to-many and one-to-many/many-to-one, the most reliable annotation will be derived from one-to-one orthologs. The reciprocal best hit method for identifying orthologs successfully identifies one-to-one orthologs between two genomes if and only if the orthologs are the top hits to each.

**Figure 3:**
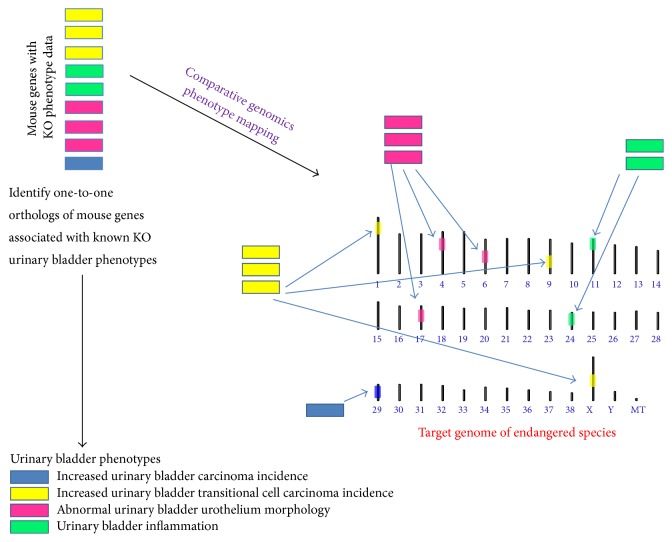
Comparative genomics approach to mapping phenotypes. Functional genomics information within a model organism genome, such as the mouse genome, can provide relevant and high confidence information about genes in other mammalian genomes. Nine mouse genes associated with phenotype data obtained from knockout (KO) strains of mice are represented in the top left corner of Figure 3. The resulting bladder cancer related phenotypes are indicated by the colors of the individual genes and the corresponding colors on the lower left corner of Figure 3. The mouse genes are mapped to their one-to-one orthologs in the target genome of an endangered mammalian species. The resulting locations of the orthologous genes are annotated with the same phenotype as the mouse gene. Blue:* increased urinary bladder carcinoma incidence*; yellow:* increased urinary bladder transitional carcinoma incidence*; pink:* abnormal urinary bladder urothelium morphology*; and green:* urinary bladder inflammation*. Once the phenotypes are mapped to the target genome, SNPs most likely to disrupt the orthologs in the endangered species (nonsense mutations and frameshift mutations) provide potential genotype-phenotype associations that mirror the phenotypes observed in knockout mice.

The tremendous wealth of mouse genomic data can be employed to enable discovery in endangered species through ortholog-based mapping (Figure 2) of mouse phenotype annotations onto endangered species genomes (Figure 3). Such an approach would provide a set of one-to-one orthologs in an endangered species with which phenotypes experimentally identified in the mouse can be associated. Combining these phenotype associated orthologs with functional genetic variation, such as missense mutations in critical residues of highly conserved domains and nonsense or frameshift mutations occurring at the N-terminal portion of protein coding genes, offers high confidence candidates for alleles likely to modulate specific phenotypes. These potential genotype-phenotype relationships can serve as the foundation for identifying members of the endangered species population that may be at risk for undesirable phenotypes.

Genomes from domesticated species, such as the dog, cat, and chicken, have also proved useful in the management of endangered species. For example, comparative genomics approaches using domestic cat MHC loci have been used to quantify MHC diversity in endangered felids including the Florida panther and the cheetah. This information provides a metric for assessing population susceptibility to emerging immunological threats such as bacteria and viruses [42]. Evolutionary conservation of short tandem repeat polymorphisms between domestic cat and cheetah facilitated the creation of PCR-primers capable of amplifying polymorphic loci in the cheetah to determine population-level genetic diversity in this endangered species [43].

Similarly, the dog genome has been used to identify short tandem repeat markers in the maned wolf, a threatened species in Brazil [44]. The red wolf is an endangered species that suffers from coyote introgression. Microsatellite markers have been developed to identify hybrids in the population and remove them in an effort to conserve the red wolf [45].

The value of model genomes for management of endangered species was exemplified in the development of chicken-condor comparative physical maps [9, 10] that were subsequently used to ultimately develop genetic tests for identifying condors at risk for producing offspring with undesirable phenotypes. The utility of comparative genomics in the conservation of endangered species will continue to be valuable, especially as new and improved tools and resources for comparative genomics are developed in the future.

## 4. Enhanced Management via Collaborative Genetic Association Studies

The identification of genetic variants within genes implicated in specific phenotypes provides a framework for identifying members of the endangered captive population that might be at risk for clinically relevant phenotypes [46]. It is important to make the distinction between validated genetic associations that are identified in typical genetic association studies and the bioinformatics based identification of genotype-phenotype candidates [47]. These candidates are not proven to be associated with the phenotypes. However, the comparative genomics association these orthologs have with their mouse counterparts offers evidence for their involvement in related phenotypes within the captive population.

Unlike domesticated species, such as cats and dogs, endangered species are at risk of extinction [48], and therefore an urgency to incorporate high confidence predictions in the management is justifiable. It is worthwhile to mention that, unlike the commercial veterinary environment where, for example, proven canine genetic diagnostics may be sold to enhance the clinical management of a pet, there is relatively little financial incentive to develop commercial genetic diagnostics for endangered species as their population size is unlikely to provide an acceptable economic return on the initial investment [49].

Another critical distinction to point out is that predicting the increased susceptibility for a particular undesirable phenotype is not the same as stating a particular member of the species which will eventually have the undesirable phenotype. Rather, it is the first step in a bidirectional communication exchange between scientists and personnel managing the species (SSP managers, zoo veterinarians, and zoo animal caretakers) (Figure 5). Management plans for endangered species rely upon multiple stake holders including veterinarians, conservation organizations, and zoos [50]. In particular, the value of the phenotype predictions is immediately realized at the level of the individual member of the endangered captive species, even if the prediction turns out to be a false positive. As an illustration, consider an endangered species in which a subset of the population has been annotated as potentially having an increased risk for certain phenotypes. If this information is made available to the zoo veterinarian, these “*at-risk*” annotated animals may receive additional scrutiny when presenting with signs associated with the predicted clinical phenotype. For example, an animal that is predicted to have an increased risk for bladder cancer may be more likely to benefit from earlier detection of disease if zoo keepers are aware that an increased rate of urination may be indicative of a problem. Regardless of whether or not the animal actually has the disease, the management is informed by the genomics knowledge, which ultimately effectively triages members of a captive population in a way that maximizes their care, health, and well-being in captivity.

Just as the sharing of genomics information from the scientist to the zoo provides a more informed management of the endangered population [3, 51], sharing of the diagnosed phenotype information from the zoo veterinarian to the scientist provides feedback on the predictions and allows the scientist to refine and ultimately identify those predicted genotype-phenotype associations for which multiple clinical assessments have provided statistically significant evidence of a true genotype-phenotype relationship. Because each zoo that contains members of a captive population can contribute to the clinically relevant phenotyping, open communication among scientists, veterinarians, and zoo staff forms the basis of a genetic association study within the captive population [52]. If every member of an endangered captive population is classified as either susceptible or nonsusceptible to a specific phenotype, based on bioinformatics based predictions of functional polymorphism consequences, it becomes possible to assess the increased relative risk (if any) associated with that particular genotype or allele.

In this particular “*clinical-management*” application of genotype-phenotype predictions, the information is only used to more effectively identify and medically manage the members of the captive population. Moreover, at this stage, the information does not need to be included in the breeding program. In fact, individual SSPs can decide when (if ever) to include predicted/verified genotype-phenotype relationships in the selection of breeding pairs. One plan for incorporating genotype-phenotype relationships in the breeding program might be based on the validation of the association based on multiple years of zoo veterinarians treating susceptible and nonsusceptible individuals. Threshold parameters for inclusion in a breeding program might include the calculated increased relative risk of the clinical phenotype associated with the genotype. Alternatively, the decision can consider the overall prevalence of the clinical phenotype within the captive population as well as the implications for population members that acquire the undesirable phenotype.

## 5. Predictive Modeling of Prevalence and Allele Frequency to Infer Relative Risk

**Figure 4:**
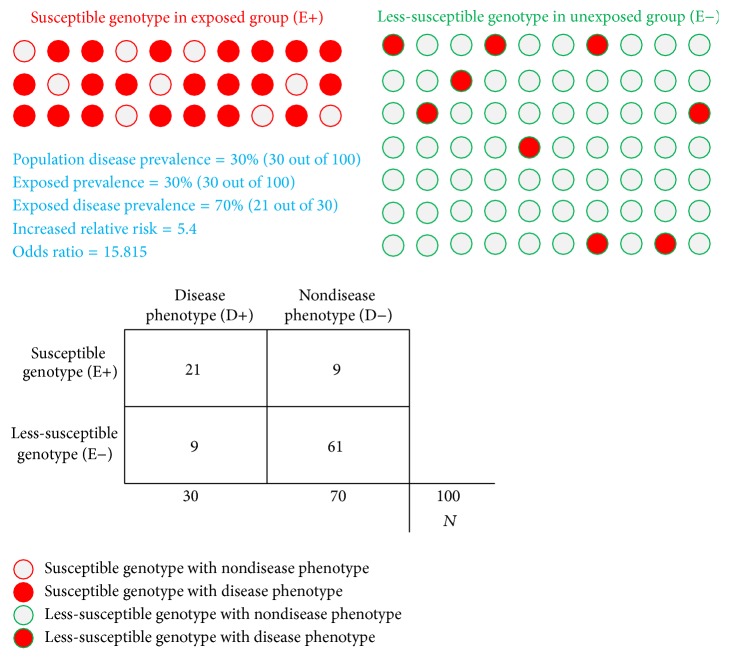
Predictive modeling of disease prevalence and allele frequencies. The value in using genomic information to predict individuals at risk for an undesirable clinical phenotype depends upon the prevalence of the undesirable phenotype (disease) in the total captive population, the prevalence of the susceptibility genotype (exposed), and the prevalence of the disease in the individuals with the susceptibility genotypes (disease in exposed group). The 2 × 2 matrix in the figure provides example values for a hypothetical endangered captive population of 100 individuals for which a disease phenotype has a population prevalence of 30%. The abbreviations “E+” and “E−” correspond to exposed and nonexposed, respectively. In this model, the exposed group has the allele/genotype(s) associated with the undesirable phenotype, while the nonexposed group does not have the susceptibility allele. Genotype-phenotype predictions, based on drastic SNP occurrence in genes associated with comparative genomics phenotype annotations derived from knockout mouse models, allow classification of members of the endangered species population into either a susceptible or less-susceptible class. Through bidirectional communication among zoo veterinarians, SSPs, zoo staff, and genomics scientists, genotype-phenotype predictions may be validated. Threshold values for increased relative risk in the exposed group, along with threshold levels of allele/genotype frequencies in the E+ and E− groups, will affect the success in employing such an approach for captive species management, as will the mode of inheritance (e.g., autosomal recessive versus autosomal dominant).

**Figure 5:**
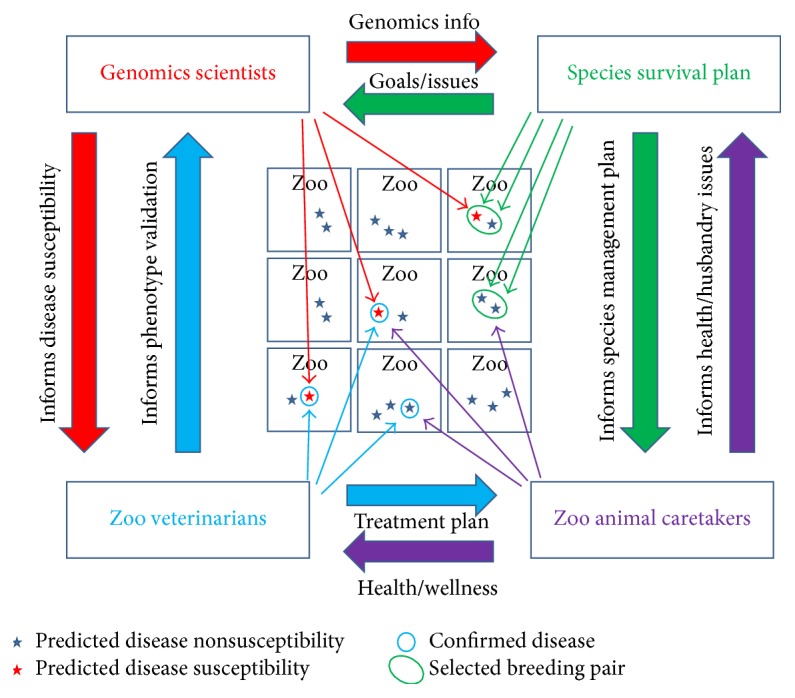
Bidirectional communication validates genomics predictions and enhances management. A communication network consisting of genomics scientists (red), species survival plan members (green), zoo animal caretakers (purple), and zoo veterinarians (blue) is illustrated schematically showing some of the possible communication paths and associated role of stakeholders in the network. Genomics scientists can provide genomics knowledge in which specific members of the endangered captive population are annotated as “*susceptible*” to an undesirable clinically relevant phenotype (red stars). Members of the population that are considered to be less susceptible are shown as well (dark blue stars). Red arrows originating from the genomics scientists and pointing at members of the captive population within zoos (red stars) represent the genomic knowledge applied to the captive population. Zoo veterinarians provide clinical assessment of phenotypes (blue circles around either red or dark blue stars) and subsequently validate genomics predictions. Species survival plan members select breeding pairs (green circle surrounding two stars) which can be informed by genomics information. For example, animals that are carriers for autosomal recessive undesirable phenotypes can be bred with partners that do not contain the undesirable allele, thereby allowing maximized genetic diversity while simultaneously minimizing the production of offspring with undesirable phenotypes. Zoo animal caretakers interact with the animals on a daily basis and provide and implement treatment and husbandry plans as well as serving as the eyes and ears for the endangered species. Purple arrows originating from the zoo animal caretakers and pointing to stars represent daily interactions of the zoo staff with the animals in the capacity of health, husbandry, breeding, socialization, and enrichment. The large colored arrows between the four stake holder's boxes represent examples of the types of bidirectional communication that can occur within the network to maximize the value of all information for the benefit of the endangered captive population.

The potential value in using genomic information to identify individuals at risk for an undesirable clinical phenotype depends upon the overall prevalence of the undesirable phenotype (disease) in the total captive population, as well as the prevalence of the susceptibility genotype (exposed) and the prevalence of the disease in the individuals with the susceptibility genotypes (disease in exposed group). As an example, consider an endangered captive population of 100 individuals, for which the overall prevalence of disease is 30%, the prevalence of the susceptibility genotype is 30%, and the prevalence of the disease within the exposed group is 70% (Figure 4). This population would have an increased relative risk of 5.4 within the exposed group compared to the nonexposed group. This model assumes that some individuals without the susceptibility genotype(s) also have the undesirable phenotype, which more appropriately models polygenic and complex genetic traits. Similarly, this model assumes that some individuals with the susceptibility genotype do not have the undesirable phenotype due to incomplete penetrance. In this scenario, the 95% confidence interval for increased relative risk of the exposed group is 3.55 to 8.35 while the overall population relative risk is 2.33. Likewise the exposed group's odds ratio is 15.82 with the 95% confidence interval of 5.54 to 45.13 and the overall population odds ratio is 2.91.

A considerable number of studies investigating the relationships between allele frequency and disease in domestic animal species have been reported recently. These reports provide a framework for considering the relationship between inherited disease phenotypes and their relationship with specific alleles and/or genotypes. For example, one study investigated a specific mutation in SOD1 and the prevalence of canine degenerative myelopathy in a population of German shepherd dogs concluded that the association of the allele with the disease supported genetic testing in clinical applications [53]. The study results showed that 8 of 50 dogs exhibited homozygosity and additional 19 dogs were heterozygous. Of the dogs homozygous for the undesirable allele that were greater than 8 years old, 42% exhibited a pelvic limb ataxia phenotype while none of nonataxic dogs were homozygous.

Another study investigating a retinal degenerative disease allele in 41 cat breeds determined that the undesirable allele frequency ranged from a high frequency of 33% to a low frequency of 2% in the 16 breeds in which it was detected. Clinical evaluations demonstrated a high correlation between the allele and the pathological phenotype. The authors conclude that, in breeds with the highest allele frequency, 7 to 13% of the individuals within the breed would be expected to develop the disease [54].

Finally, a 2010 study of polysaccharide storage myopathy in horses, caused by a mutation that had been identified in over 20 different horse breeds, determined that the prevalence of inherited susceptibility to the undesirable phenotype varied within these breeds from high prevalence of 62% to low prevalence of 0.5%. The management implications for this genetic information included (1) strategies for breed associations to consider screening for this specific mutation and (2) use of the undesirable allele as an “alert” for veterinarians to more closely evaluate a horse for myopathy related clinical signs such as altered gait, muscle pain, and rhabdomyolysis [55].

In these scenarios, the relationship between allele frequency and disease prevalence (or incidence) depends upon the mode of inheritance. In practice, the threshold limit of minor allele frequency in the population as well as the population disease prevalence will affect the ability to successfully employ genotype-phenotype predictions in endangered captive populations. Since the clinical assessment of undesirable phenotypes is required to validate a particular prediction, the frequency with which members of the species are clinically assessed each year will contribute to the rate at which validation can occur. Ultimately, for some phenotypes in certain species, this approach may not be viable; however, true value in this approach lies in cases where an autosomal recessive genotype-phenotype relationship can be established in the endangered captive population. This knowledge can be applied to more effectively manage breeding by selecting breeding pairs that minimize the production of the undesirable phenotype by allowing only propagation of carriers. This “*carrier-only*” approach can maximize genetic diversity in the captive population by including affected individuals in breeding programs, especially if they are prolific breeders, while minimizing the production of affected offspring produced by these carriers of undesirable traits.

Ultimately, genomic resources coupled with per-individual genomic fingerprints can lead directly to highly improved endangered species management. For example, deleterious mutations can be identified by examining how each SNP and indel impact the gene in which the mutation is found. Highly impactful SNPs, such as those interrupting a coding region, can be mapped on a per-individual basis. If phenotypes are available in sufficient numbers of individuals, these mappings can be used to map genomic traits to the phenotype of interest using techniques such as whole genome association studies (GWAS). Where phenotype data is not sufficiently available, these mappings can be used to infer likely nondesirable configurations and used in a similar fashion on a per-individual basis. For example, if a gene has been identified and predicted to provide a phenotypically relevant function, variation mappings across individuals can be used to separate those likely healthy from those likely unhealthy. Such results can be directly applied to development of individualized health plans and to guide breeding strategy.

## 6. Paradigm Shift in Management Culture

The challenge in effectively applying genomics knowledge to the management of endangered captive species arises through a combination of various stake holder opinions and assumptions coupled with the limitations posed by a small highly prized population, for which individuals are easily perceived as devalued via undesirable health labels (i.e., phenotypes). Part of the issue lies in the perceived value or quality of a particular member of the species. For example, if a zoo seeks to participate in an SSP's breeding program for a particular species, acquiring an animal that may produce less desirable offspring may be problematic. Currently, an animal may be included or excluded from a breeding program based on the SSP's determination of its impact on genetic diversity of the population as determined by mean kinship calculations [56]. It also may be excluded from breeding due to the presence of an undesirable phenotype or chromosomal abnormalities [57]. If genomic-based information was to identify a greater number of individuals with a genotype linked to that phenotype, some stakeholders fear that more genetically valuable animals would be excluded from the breeding population. However, it is not required (or even preferable) to remove such “undesirable” genetic variants from the population. The power of genomics in captive endangered species may be to identify targeted breeding strategies that would minimize the impact of such variants phenotypically while still maintaining acceptable genetic diversity. In fact, removing all individuals with undesirable phenotypes from the breeding pool can result in considerable loss of genetic diversity and may even permanently remove low frequency polymorphisms of high value from the endangered captive population.

In order to maximize the value of genomics information in the management of captive endangered species, all stakeholders must appreciate the utility genomics knowledge can bring to management decisions. Successful application of genomics information in captive management will require a shift in management culture to integrate the use of the burgeoning information from the variety of resources now available to those entrusted with the care of these animals. Just as important, in order to achieve the maximum benefit that genomic information can offer, bidirectional communication must occur between caretakers managing the health of the endangered animals and scientists mining the genetic information. A shared database containing phenotypic information for each species, including physical characteristics and variations, physiologic parameters and ranges, and disease prevalence and expression, would be invaluable for functional genomics investigations. Similar databases have been previously developed for use in the breeding of transgenic mice [58]. Ultimately, conservation efforts will benefit from a prioritized commitment to value shared knowledge among all stakeholders.

The benefits of using genomics can enhance the role of zoos and SSPs by giving us knowledge about the health and attributes of a specific animal much earlier in that animal's life than our current and traditional process of waiting for and observing traits and symptoms only when they become physically apparent [59]. This can be tremendously beneficial to breeding programs as more will be known about each animal at a much earlier point in their breeding career, allowing for healthier matches and better long term genetic outcomes for the species.

Endangered species are the ideal species for the use of genomics knowledge for a number of reasons. There is tremendous public interest in and support for endangered species and the programs to preserve them. Therefore utilizing new science-based approaches, especially those that have no negative impact on the endangered individuals themselves, will enhance the public standing of the facilities and groups involved. By illustrating the value of genomics information in endangered species and the management decisions their protection requires, we can shift the paradigm for many conservation stakeholders and ultimately benefit many species fairly rapidly.

## 7. Conclusion

The successful conservation of wild and captive endangered species will undoubtedly evolve as genomics knowledge becomes more widely applied to management decisions. Functional genomics, as applied to conservation genetics in animal populations, entails understanding how the genome of an individual animal or the collective genomic properties of a population influences the well-being and survival of that individual or population. Genomics knowledge, such as sequence data, polymorphism data, and gene expression data, provides an unprecedented opportunity to consider the entire genome to better understand how genetics contributes survival for a particular species. Just as comparative physiology and comparative anatomy offer a context for appreciating the mechanisms underlying variation in form and function, comparative genomics aids in elucidating conserved and divergent genetic mechanisms associated with specific phenotypes. Of particular interest are comparative genomics approaches that can efficiently and effectively identify functionally important genomic regions with implications for health and disease. Model organism genomes, from animal species, such as the mouse, dog, and cat, have already contributed to advances in the management of endangered felids and canids. As bioinformatics algorithms and pipelines become more sophisticated, the identification of genetic variants within genes implicated in specific phenotypes will facilitate identifying members of the endangered captive population that might be at risk for clinically relevant phenotypes. Such individuals can be given additional medical scrutiny to maximize the opportunity for early detection of clinically important conditions. Additionally, this genomic information can be used to better manage breeding programs, for example, to limit the production of homozygous autosomal recessive undesirable phenotypes. However, to most effectively maximize the value of genomics information in the management of captive endangered species, all stakeholders must appreciate the utility genomics knowledge can bring to management decisions. Successful application of genomics information in captive management will require a shift in management culture to integrate the use of the burgeoning information from the variety of resources now available to those entrusted with the care of these animals.
